# Development of a Green Roof Environmental Monitoring and Meteorological Network in New York City

**DOI:** 10.3390/s90402647

**Published:** 2009-04-15

**Authors:** Stuart R. Gaffin, Reza Khanbilvardi, Cynthia Rosenzweig

**Affiliations:** 1 Center for Climate Systems Research, Columbia University, New York, NY 10025, USA; E-Mail: crr2@columbia.edu; 2 Department of Civil Engineering & Earth and Environmental Sciences, City College of New York, New York, NY, USA; E-Mail: rk@ce.ccny.cuny.edu

**Keywords:** Urban heat island, green roofs, combined sewer overflows, energy balance, urban vegetation, mitigation, building energy

## Abstract

Green roofs (with plant cover) are gaining attention in the United States as a versatile new environmental mitigation technology. Interest in data on the environmental performance of these systems is growing, particularly with respect to urban heat island mitigation and stormwater runoff control. We are deploying research stations on a diverse array of green roofs within the New York City area, affording a new opportunity to monitor urban environmental conditions at small scales. We show some green roof systems being monitored, describe the sensor selection employed to study energy balance, and show samples of selected data. These roofs should be superior to other urban rooftops as sites for meteorological stations.

## 1. Introduction

Urban heat island (UHI) research is undergoing a renaissance of interest as a climate issue. As one of the oldest observations in climatology, much research had already been done on the phenomenon by the 1960’s and 1970’s [1]. Now, as the specter of global warming looms ever larger, urban heat management has taken on a new imperative, as excess urban heat will only exacerbate the problems of global warming for residents. Indeed, heat island intensity in many urban areas is already comparable to the amount of warming expected regionally over the next century [2]. Secondly, mitigating urban heat sources is an adaptation strategy that will be equally important for overall global warming mitigation in residential areas worldwide. Thirdly, urban population increases are a pervasive demographic settlement trend that will be ongoing over the next century. Recently, urban population surpassed non-urban population worldwide. United Nations projections suggest that the percentage of world population living in urban areas will grow from 50% currently to 70% by 2050 [3]. In terms of absolute numbers, urban population will grow from ∼3.33 billion today to ∼6.4 billion in 2050, about a 90% increase. Put another way, the number of people living in urban areas in 2050 will be close to the entire world population today. These estimates underscore the practical relevance of urban environmental mitigation measures as most of humanity will be living in cities.

There can be little question about the broad array of urban resident issues that are negatively influenced by UHI. These include: extreme peak energy demand, heat wave stress and mortality, poor air quality, local ecological impacts, thermal shocks to waterways following rainfall, and even impacts on urban precipitation [4,5].

### 1.1. Urban Runoff Islands:

A less widely-appreciated urban environmental problem than UHI is excessive storm-water runoff. This phenomenon, similar to urban heat, has its origin in the preponderance of impervious surfaces in cities. It follows even modest rain events wherein a wave of runoff water from streets, buildings and rooftops, hits the municipal sewer system with which it is typically combined, but which has limited capacity because of water treatment facility capacity. As a result, the excess water mixed with sanitary water from buildings is diverted into local waterways, leading to a “combined-sewage-overflow” (CSO). In the same sense that dark, impervious urban surfaces for streets and rooftops are efficient solar collecting surfaces leading to ‘urban heat islands,’ they are also efficient rainfall collection surfaces creating ‘urban runoff islands.’

### 1.2. Green Roofs:

Green roofs (living eco-roofs) are garnering increasing attention within the United States as a valuable urban mitigation technology, although they have been popular in Europe for many decades. They are engineered systems typically consisting of a series of layers including (from bottom to top): (i) a waterproof and root-proof membrane, (ii) a drainage layer for excess water; (iii) a filter fabric; (iv) growth medium - which is not soil but engineered lightweight granular medium typically consisting of expanded shales and clay minerals; and (v) plants, available in a growing variety, but typically sedums [6]. One of the strongest arguments favoring widespread green roof adoption in cities is their *versatility* for addressing multiple concerns including heat, runoff and other issues such as the need to restore ecological habitat and green space [6].

A useful classification of green roofs is often made into either shallower “extensive” or deeper “intensive” systems, where shallower and deeper refer to growing medium depth. While an exact depth demarcation does not exist, generally extensive roofs refer to those less than 15 cm, while intensive roofs are equal or greater than 15 cm [12]. The depth variation also strongly dictates plant options, allowing for a much greater plant palette for intensive roofs. It is likely that deeper systems confer greater environmental performance including temperature reductions and water retention capacity and it also may be true that native plantings improve these benefits as well. But this remains a question for field-analysis.

Figure 1a shows a 1-year old sedum 10 cm extensive green roof that is currently part of our monitoring network. The plants were installed as small plugs in September 2007 and have filled in robustly since then. More detailed information and photographs about this particular project are available at: www.ecfs.org/about/sustainablefieldston/roof.aspx Figure 1b shows a second sedum roof project installed at Columbia University that is also approximately 1-year old. The plants in this case were pre-grown prior to installation and delivered as mats. In both cases the plants are thriving in what would normally be a harsh environment, and with relatively little maintenance.

With respect to runoff, green roofs can be viewed as ‘leaky’ water storage tanks, that also evaporate large amounts of water vapor. A typical 10-cm green roof medium, when fully saturated can hold ∼ 1 gallon of water per square foot. In New York City, rooftops comprise approximately 13 % of the total land surface area, which translates into about 40 square miles of rooftop surface for the entire metropolitan region, underscoring a vast potential water retention and detention capacity that is not currently being exploited. The ‘soil’ water storage, plus vegetation further leads to evapo-transpiration which results in latent heat cooling. We will be showing data in this communication on the temperature reductions on green roofs as compared to traditional rooftop membranes.

### 1.3. Green Roofs As Better Urban Weather Station Locations?

There is still much to be learned about urban climatology too. For example, are there significant persistent micro-variations in UHI intensity within an urban landscape. In principle, one would assume yes, but convincing data on this is still not elementary to come by, without a good network of observation stations.

There are well-known challenges to locating weather stations in urban areas. Instrument security at ground level is one issue. Also ground level siting, even if secure will present problems with respect to nearby building obstructions and biases from extraneous heat sources and wind distortions (air conditioning, vehicular effects, building facades that affect winds, etc).

As for air temperature measurements, the National Weather Service [7] guidelines on temperature measurements include the recommendation that “…the sensor should be at least 100 feet from any paved or concrete surface” [7].

Campbell Scientific Instruments, a major supplier of dataloggers for meteorological and field sensors offers the following guidelines on siting and exposure of weather stations:

“…Typically the site should represent the general areas of interest, and be away from obstructions such as buildings and trees… Wind sensors should be located over open level terrain…The open areas should be covered by short grass, or where grass does not grow, the natural earth… Avoid … rooftops.” [8]

The proscription against paved surfaces and rooftops -- and for grass or natural soil cover -- is because of the extreme temperature ranges that traditional rooftop membranes (dark bituminous) will experience. Traditional membrane temperatures greatly exceed air temperatures during the day but are also much lower at night. This heat source and sink can clearly bias nearby air temperature readings.

**Figure 2.**
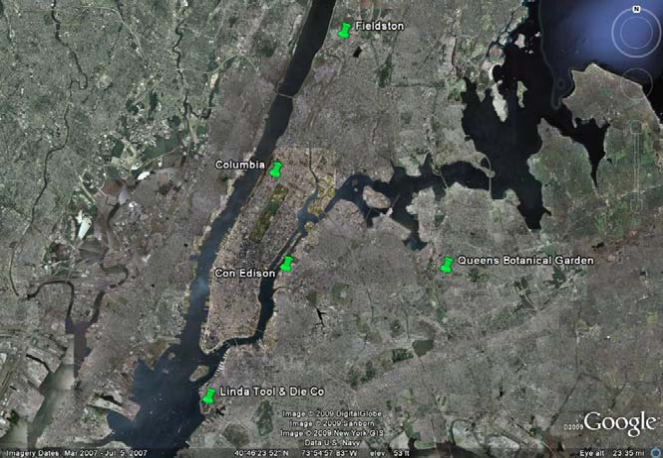
Location map showing research stations that currently comprise our green roof meteorological network. The green roof photographs shown in Figures 1a and 1b correspond to the “Fieldston” and “Columbia” map locations respectively. These two stations are separated by a distance of ∼10 km. Three of our research stations are extensive green roofs (Fieldston, Columbia, Con Ed) with medium depths of 10-cm or less. The two other stations are semi-intensive with medium depths of 15-cm or more.

To investigate such issues, we have been deploying weather station and green and control roof monitoring equipment on a growing number of sites within the New York metropolitan region. Currently our network consists of five separate locations covering major boroughs of New York City (Figure 2). In the next section we describe the general sensor selection and deployment and show selected data for some basic metrics.

## 2. Selected Sensors and Selected Data

The physical considerations that govern our choice of monitoring equipment for green roofs, control roofs and weather station sensors are to collect sufficient data to: (1) perform a surface energy balance and (2) perform a surface water balance. These two balances are fundamental boundary layer conservation statements that underpin climate modeling, for example. They can be done at various levels of complexity and rigor, given that a finite depth porous medium and plant cover are included [9].

**Figure 3.**
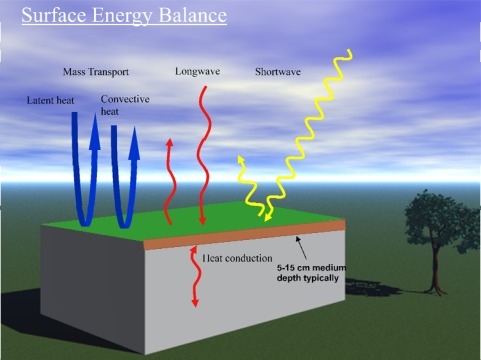
Simplified surface energy balance diagram for a green roof illustrating the major energy fluxes that need to be measured or estimated.

Figure 3 conceptually depicts a simplified surface energy balance for a generic green rooftop system. The diagram is simplified with respect to the energy within the green roof layers. Given that a green roof with medium has a finite thickness, typically 5–15 cm, more refined energy balance would treat sub-layers for each unit depth, providing data for a heat flow analysis within the growth medium. Also the plants themselves have a complex leaf and stand geometry that can be analyzed at increasing levels of detail. A similar water balance diagram can be drawn which includes precipitation, runoff, soil moisture content and evapo-transpiration (The focus of this paper will be on energy balance considerations for green roofs. Water balance analysis on green roofs will be the focus of a subsequent publication).

Ideally, monitoring equipment is chosen to permit a reasonably accurate estimate for each of the arrows – energy fluxes per unit area – shown in the figure. For some of these fluxes appropriate sensors exist and it therefore relatively straightforward to monitor them. Others, particularly the mass transport latent and sensible fluxes, present greater challenges.

### 2.1. Shortwave Fluxes and Albedo

**Figure 4.**
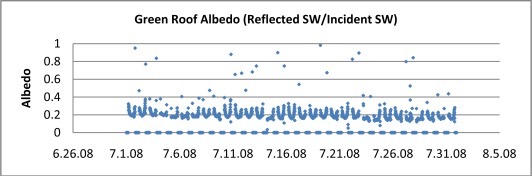
Albedo for the green roof shown in figure 1a during July 2008. The pronounced diurnal cycle is due to the change of reflectivity with incidence angle.

Shortwave radiation is usually defined as the bandwidth comprising from 300 to ∼2,800 nanometers [1]. For our green roof applications we have been using a Kipp and Zonen CMP3 pyranometer, which has this same measurement band [8]. With two of these sensors placed back-to-back, simultaneous downward and upward fluxes of shortwave radiation can be monitored and thus produce albedo for the test surface over which the fluxes are being recorded. Although quite easy to monitor, incident shortwave radiation is not as commonly recorded on weather stations to near the extent of temperatures, winds or other meteorological data. Figure 4 shows albedo data for the green roof in Figure 1a for the month of July 2008, near the time the photograph was taken.

**Figure 5.**
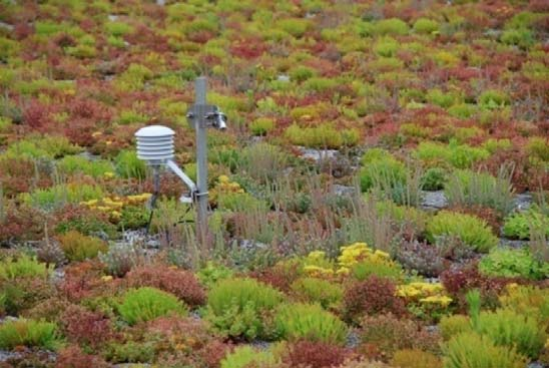
Close-up view of 1-year old sedum plant cover on the green roof in figure 1a. The plants began as nearly invisible plugs and fill in robustly over time. Also shown is a typical green roof monitoring stand that supports various surface and sub-surface sensors included 1-foot air temperature and relative humidity, a surface infrared radiometer to measure leaf temperatures, buried thermistors for temperatures at different vertical horizons and soil moisture probes (photo: S. Kedia).

A pronounced diurnal U-shaped cycle for albedo is evident. Albedo is a minimum at noon when incidence angle is minimized. The U-shape is largely due to the change in solar incidence angle during the day, affecting surface reflectivity, however other factors, including perhaps leaf responses may be playing a role. The time averaged albedo for the July data is 19.6%. By comparison Oke [1] presents diurnal data for short turf grass albedo ranging from 25–30%, higher than seen for the sedum plants on green roofs. A close up picture of the sedum leaves is show in Figure 5. The taller stand and complex shape of these plants may well be more effectively trapping incident shortwave radiation than typical turf grass.

### 2.2. Longwave Fluxes: Downwelling and Upwelling

The upwards and downwards longwave energy fluxes are arguably the easiest to parameterize with respect to observed temperatures. If the surface temperatures and emissivity (ɛ) of the surface are known then the upwards longwave radiation is simply given by the Stefan-Boltzmann law for grey-body radiation:

(1) $$\mathrm{LW}↑=ɛ\cdot \sigma \cdot T_{\mathrm{surf}}\;{\left(t\right)}^{4}$$

Emissivity for most natural surfaces has a relatively small range from 0.82–0.99 [1]. Energy balance models commonly assume an emissivity of 0.9 – 0.95. Similarly, a good deal of empirical work has been done to correlate downward longwave radiation from the atmosphere with surface temperatures and relative humidity [10]. These assume the same Stefan-Boltzmann law relation of equation (1) and then develop empirical formulae for the atmospheric emissivity as a function of surface temperatures, relative humidity or both. Under cloudless conditions these empirical equations have a relative accuracy of 5%. However, sensors are increasingly becoming available to measure all four SW and LW fluxes in one compact unit. For example, Kipp and Zonen provides a net radiometer (CNR2) consisting of back-to-back pyranometers (for net SW) and pyrgeometers (for net LW) and produce a net reading of radiation flux received at the surface [8]. The instrument is typically mounted 1.5 meters above the surface.

**Figure 6.**
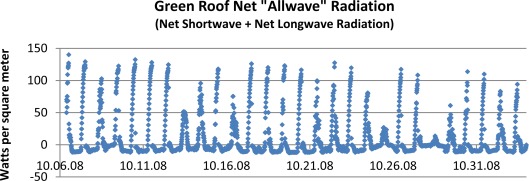
Net “allwave” radiation being absorbed by a four-inch green roof system within our network. The data time interval is 15 minutes. For much of the diurnal cycle, net allwave radiation is negative except for the mid-day hours of peak solar radiation.

Figure 6 shows one month of data on net radiation on one of our 4-inch deep sedum roofs located at the “Con Edison” site shown in Figure 2. The noon peaks in radiation correspond to the dominance of downward incident solar radiation. At night, in the absence of sunlight, net longwave cooling to space occurs. Interestingly, for much if not most of the daily cycle, net allwave is negative. The effect of clouds on net surface radiation is discernable near the dates of 10/25/08 and 10/28/08 in the chart. Here it is seen that night-time radiative loss is near zero showing the effect of increased downward radiation from clouds.

The three remaining energy fluxes not accounted for in this graph are: (i) sensible and (ii) latent heat losses and (iii) heat flow within the green roof medium [11]. In general, during the day, latent and sensible heat losses are offsetting the positive radiative flux, thus slowing the buildup of heat and temperatures within the green roof layers. At night they are generally adding to the radiative cooling fluxes and accelerating the heat loss and temperature reductions [11].

### 2.3. Sensible and Latent Heat Fluxes

There is a vast literature on atmospheric boundary layer transport processes that govern surface sensible and latent heat fluxes, but which is beyond the scope of this paper to review. A range of simplifying assumptions need to made to obtain tractable, empirical formulae for these fluxes based on data that is reasonably easy to monitor. One approach is known as the “aerodynamic profile method” – a version of flux gradient theory [1]. This approach assumes that there is neutral stability, steady state, constancy of fluxes with height and similarity of all transfer coefficients. With these assumptions it can be shown that sensible heat flux at a surface is given by:

(2) $$Q_{\mathrm{sensible}}=-C_{a}\;k^{2}\;Z^{2}\left(\frac{\Delta \bar{u}}{\Delta z}\cdot \frac{\Delta T}{\Delta z}\right)$$

where, C_a_ is atmospheric heat capacity, k is von Karman’s constant (∼0.4), z is the vertical reference height for wind speed and temperature measurements, u is horizontal windspeed and T is atmospheric temperature at the reference height, z. A similar theoretical equation can be developed for latent heat fluxes due to evapo-transpiration:

(3) $$Q_{\mathrm{latent}}=-L_{v}\;k^{2}\;Z^{2}\left(\frac{\Delta \bar{u}}{\Delta z}\cdot \frac{\Delta \rho_{v}}{\Delta z}\right)$$

where, L_v_ is the latent heat of vaporization and ρ_v_ is density of water vapor in the ambient air parcels, which can be related to relative humidity. With sufficient data on near-surface windspeeds, temperatures and vapor densities, vertical gradients in equations (2) and (3) can be approximated using reference height measurements of horizontal windspeed, temperature and relative humidity -- common weather station metrics. Since the other constants and dimensions are known, sensible and latent heat fluxes can be estimated.

### 2.4. Surface Temperatures

For surface temperatures, surface infrared radiometers invert equation (1) to obtain radiometric temperatures. This is particularly useful for monitoring green roof leaf temperatures which have a complex geometry (Figure 5). Therefore in all of our installations we routinely employ numerous surface infrared temperature sensors. Similarly, relative humidity sensors are easy to deploy at near surface environments to allow for vapor density calculations. An alternative approach for latent heat involves the “Bowen” ratio method which assumes latent heat is proportional to sensible heat flux [11]. In addition we have included “control” non-green roof sensors in all or our applications. The control roofs are often the dark bituminous asphalt roof. A surface radiometric temperature sensor is usually sufficient although vertical profiles of air temperature are useful for assessing sensible heat fluxes.

**Figure 7.**
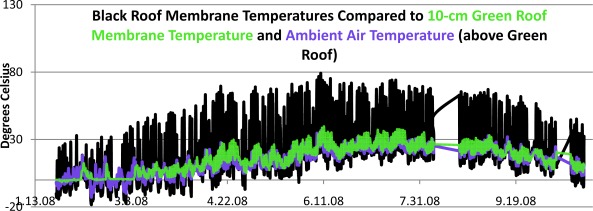
Comparative surface membrane temperatures on a black roof compared to the 10-cm sedum green roof of figure 1a. Also shown in blue are the ambient air temperatures monitored above the green roof. The dramatic reduction in temperatures on green roofs is evident. The green membrane temperatures are close to ambient air temperatures.

Figure 7 shows a graph of ambient air and membrane temperatures on the green roof pictured in figure 1a, compared with membrane temperatures on a nearby black roof. The green temperature line corresponds to the green roof membrane below the growing medium at a depth of 10-cm. Many features are worth noting in this graphic. Most prominently the extreme temperature cycles on standard black roof membranes are evident, with summertime peaks approaching 80° C (176° F). Such temperatures exemplify rooftops as major urban heat sources. Also notable is the rapid nighttime cooling of the black roof membrane, significantly below the ambient air temperatures. These black roof diurnal temperature swings, approaching 70° C, create thermal expansions and contractions which are a major cause for rooftop membrane deterioration over time.

The green roof membrane, in contrast, has a greatly suppressed thermal cycle owing to the cooling properties of green roof plants and growth medium. Among other benefits the green roof has almost eliminated the rooftop as an urban heat source. With respect to urban rooftop weather station siting, the green roof is a dramatically better location for ambient temperature readings. The absence of extreme temperature cycles also helps prove why green roofs are expected to outlast traditional membranes by 2 or more times, since the accompanying thermal expansions and contractions are greatly reduced [12].

### 2.5. Heat Flow

We have taken two approaches to estimating heat flow on green roofs. Since these roof systems employ a porous growth medium, in principle, they permit the use of direct heat flow sensors which are available from soil science applications [8]. An alternative approach is to measure the vertical temperature profile within the medium and estimate heat flow from temperature gradients. However this requires dealing with the challenges of estimating the changing medium thermal diffusivity coefficients as water and air content change. Figure 8 shows one year (2008) of hourly heat fluxes within the medium of the 2-inch sedum green roof in Figure 1b. Positive heat flows correspond to downward heat flux during the day, driven by higher surface temperatures from solar heating. Negative heat fluxes are occurring during the night as the temperature gradient reverses. During the course of the year the total net flux of heat from this roof was −43.9 KWhr/m^2^. In other words, there was an annual net loss of heat from the building to the atmosphere, due to a combination of building heat loss to the atmosphere and incident atmospheric radiation that has not been transferred successfully through the roof insulation. The total green roof area for this project was approximately 350 m^2^. Thus the total net annual heat loss through the roof was −15.3 MWhr. However, interpreting this net flux in terms of building energy use will be challenging.

### 2.6. Urban Heat Islands at Small Spatial Scales

Urban heat island (UHI) analysis typically entails comparing urban temperature records with those of stations far removed from the urban core, in surrounding suburban or rural locations. New York City has a well defined UHI which has been quantified by a number of analyses ([13]–[15], [2]). These studies have shown a predominantly nocturnal heat island, with an annual average value of approximately 2.5°C [2] where urban temperatures are compared with rural temperatures approximately 50–100 km away.

Figure 9 shows the air temperature difference between the two green roof sites shown in Figure 1. These two sites are located due North-South of each other and are separated by a notably short distance of only ∼10 kms. The northern site in the Bronx (Figure 1a) is a fairly heavily forested area directly North of Manhattan Island. The local tree canopy is partially evident seen in the figure. By contrast the Manhattan site near Columbia University is typical dense urban terrain, with little street vegetation, as seen in Figure 1b. Despite their proximity, the air temperature difference between these two locations is quite strong and averaged 1.45 °C for the period shown. There is evidence of seasonality too with the Spring and Summer seasons showing increased differences.

The temperature difference peaks, at times 3–5 °C, tend to be nocturnal as has been seen in other studies of Manhattan’s heat island hourly variation [2]. One can note the rarity for when the Manhattan site cooler than the Bronx site. As to the probable causes of this micro-urban heat island difference, the much denser vegetation cover in the Bronx site is likely playing a key role. In addition, the reduction in anthropogenic heat sources there is also probably a factor. Micro-urban heat island variations such as this are of practical interest. If the causes are indeed due to vegetation cover, they demonstrate a significant UHI mitigation potential for tree-planting and green roofing programs. Further, if hotspots within cities can be identified this would help prioritize such programs as well as inform peak energy management data, for example.

## 4. Conclusions

Green roofs are versatile systems that are becoming increasingly important to address a number of urban environmental concerns. Chief among these are urban heat and stormwater runoff reduction. However a number of additional benefits will accrue both to the individual building owners and the public. These include heating and cooling building energy reductions, greatly extended roof service life, enhanced building amenity value, increased urban green space and ecological habitat creation, with the potential for preservation.

Given the vast land surface area that rooftops typically comprise in cities, the collective benefits from widespread adoption are considerable. For New York City we estimate that rooftop surface area probably equals 20–30 times the land area of Central Park. As protected and secure spaces, rooftops have many advantages as a site for urban vegetation. Presently traditional rooftop membranes have only adverse environmental impacts and are blighted and neglected spaces as well.

Collecting data on green roof performance is an important scientific goal for the near term as urban policy makers begin to compare the costs and benefits of different mitigation technologies. Other technologies exist to address urban heat and runoff, some proven and others in development stages only. We largely consider green roofs a proven technology, however they are still expensive compared to traditional rooftop membranes. Also there are many different green system designs including variations in layers, plants, medium, system delivery and installation. These alternatives also have different maintenance requirements and costs.

In this paper we have begun to develop a network of green roof research stations that will allow us study system differences as well as to create a new prototype of urban meteorological network. The present network is only in the early stages and we have not attempted a definitive analysis of the data being collected here, which is voluminous. For example, quantifications of the sensible and latent heat fluxes will require theoretical treatments using primary meteorological data. Rather we have sought to illustrate some of the basic system parameters that are easily measured for the purpose of performing energy and water balances. The interesting preliminary findings include: (i) the distinctive diurnal cycle for albedo. Albedo is clearly a dynamic property of land surfaces although it is often treated as a constant in boundary layer climate models. The albedo for sedum green roofs (∼20%), although much higher than dark roof membranes, is not sufficient to explain the lower temperatures we observe on green roofs. The additional cooling is due to evapo-transpiration, or latent heat, as the authors have shown in other work [2]. (ii) The temperature cycle amplitude reductions on green roof surfaces are dramatic and remove any temperature bias that would normally occur if an urban weather station were placed on a traditional dark impervious roof membrane. These temperature reductions also have implications for reduced building cooling and heating requirements both in the summer and winter seasons. The temperature reductions also support a purported extension of green roof service lifetime over traditional membranes. (iii) The presence of a growth medium comparable to soil on green roofs means that soil heat flux plates can in principle be used, although this will require further study as the shallow depths are unusual for soil applications. The presence of such a heat flux place of rooftops may afford an unusual opportunity to analyze heat flow energy losses from buildings. (iv) Our station network allows us to study micro-climate variations within the metropolitan region. We are beginning to see interesting data that indicates UHI can vary significantly on scales of kilometers. There are a number of practical applications to such data such as energy management and heat island mitigation policy. Our analysis of the water balance and retention properties of green roofs is only in its early stages as well and will be the subject of a subsequent report.

## Figures and Tables

**Figure 1. (a):**
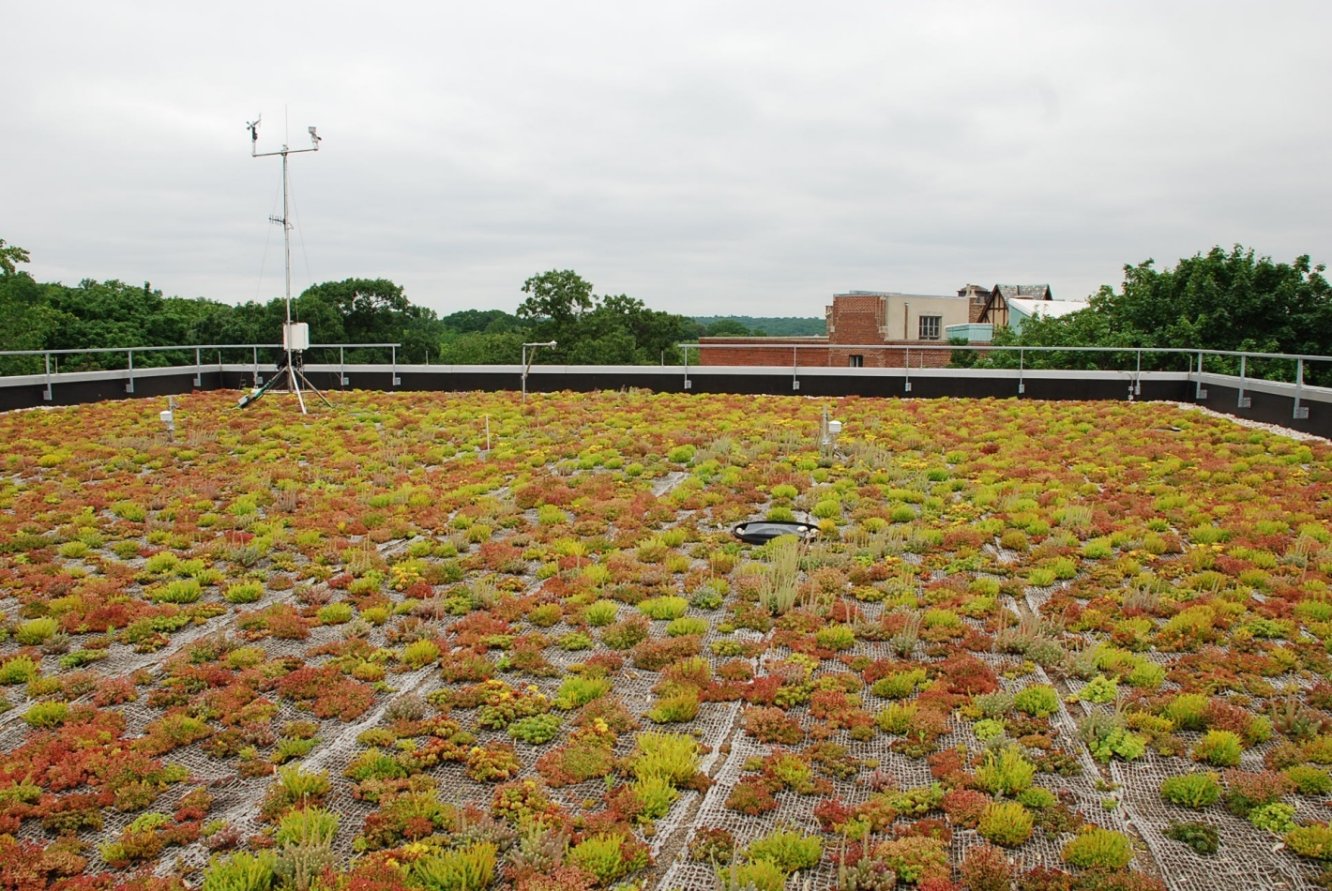
The Bronx, NY green roof research that is one of our network sites. It consists of 10-cm medium planted with mix of 6 sedum species of plants. Sedums are very drought, heat and cold tolerant, as well as being low maintenance. A jute cloth is visible and was used to provide ballast for the lightweight medium during the initial plant establishment period and also improve moisture retention. This roof is one-year old and the plants will continue to fill in over time. Weather station and *in situ* green roof monitoring equipment are visible in the background (photo: S. Kedia).

**Figure 1. (b):**
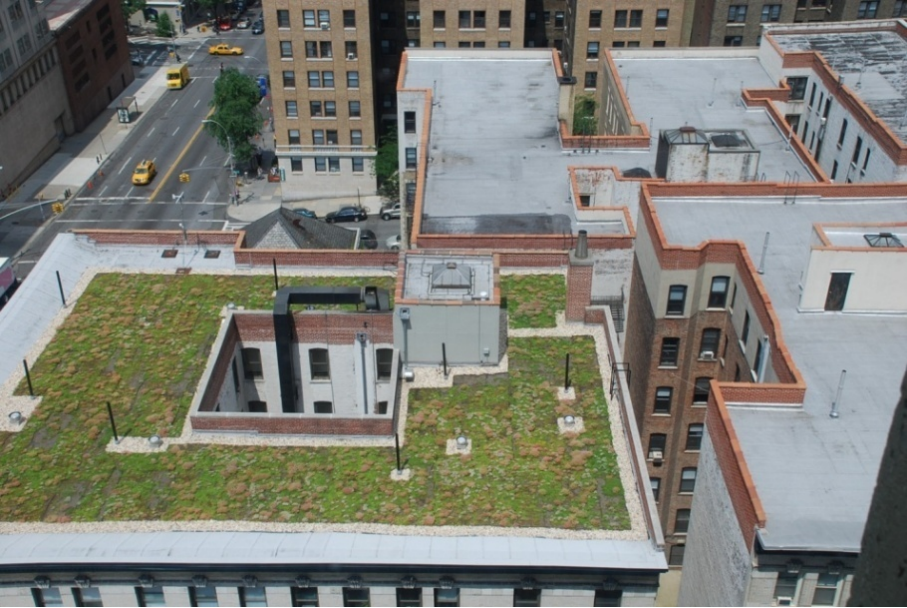
Columbia University, New York green roof research station. This green roof is a thinner ∼5 cm deep system planted with mix of sedum species of plants. The roof is one-year old and is ∼350 m^2^. Weather station and *in situ* green roof monitoring equipment are partially visible in the background (photo: S. Kedia).

**Figure 8.**
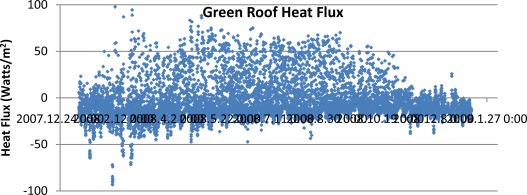
Heat flux through the green roof shown in Figure 1b. The heat flux is being measure by a “Hukseflux” soil heat flux plate [8]. Negative heat flow is towards to the atmosphere and positive is downward towards the building roof layers. By far, more heat is being lost to the atmosphere annually, which is due to a combination of building heat flow upwards and through the roof layers and any incident radiation energy that has not been transported downward through the building insulation layers.

**Figure 8.**
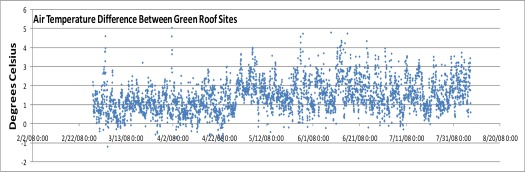
Hourly air temperature differences between the two green roof projects shown in figures 1a and 1b. The two sites are only separated by ∼10 kms, with the station in figure 1a being due North and located in a heavily forested area of Bronx, New York (Figure 2). The Columbia University station by contrast is located in a typical dense urban area of upper Manhattan, with little street tree cover and vegetation.
